# P-2053. Clinical Outcomes and Severity of Hospitalized COVID-19 Patients Among Partially and Fully Vaccinated Patients in Louisville, Kentucky

**DOI:** 10.1093/ofid/ofae631.2209

**Published:** 2025-01-29

**Authors:** Waqas Shahnawaz, T’shura Ali, Nouman Shafique, Jafir Wakeel, Daniya Sheikh, Vidyulata Salunkhe, Steven Gootee, Forest W Arnold

**Affiliations:** University of Louisville, Louisville, Kentucky; University of Louisville School of Medicine, Louisville, Kentucky; University of Louisville, Louisville, Kentucky; University of Louisville, Louisville, Kentucky; University of Louisville School of Medicine, Louisville, Kentucky; University of Louisville School of Medicine, Louisville, Kentucky; University of Louisville School of Medicine, Louisville, Kentucky; University of Louisville School of Medicine, Louisville, Kentucky

## Abstract

**Background:**

Despite COVID-19 vaccine availability, reducing disease severity and hospitalizations remains crucial. Persistent vaccine hesitancy necessitates studies comparing outcomes between partially and fully vaccinated individuals. Our study aims to evaluate clinical outcomes among hospitalized COVID-19 patients, distinguishing between partial and full vaccination.

Multivariable logistic regression analysis for ICU admission among fully and partially vaccinated patients with COVID-19 CAP

**Figure:**
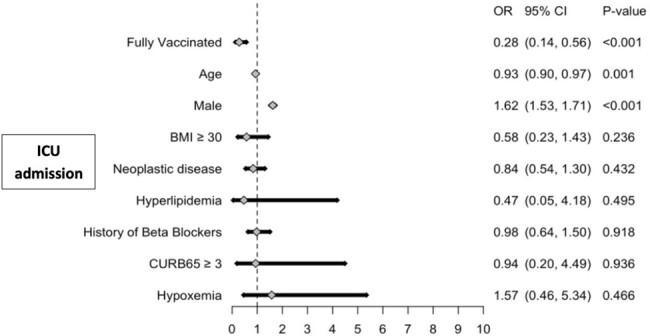

**Methods:**

We conducted a retrospective cohort study among hospitalized COVID-19 patients with community-acquired pneumonia (CAP) in Louisville, Kentucky. Clinical outcomes, including in-hospital mortality, cardiac events, ICU admission, and use of mechanical intubation and ventilation, were assessed. Statistical analyses included univariate and multivariable logistic regression to compare outcomes between vaccination statuses.

Multivariable logistic regression analysis for Mechanical intubation and ventilation use among fully and partially vaccinated patients with COVID-19 CAP

**Figure:**
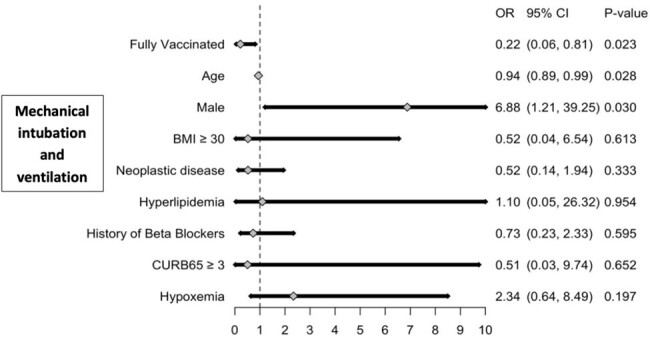

**Results:**

Of 142 eligible patients, 87% were fully vaccinated, with 13% partially vaccinated. The fully vaccinated group had less patients with a CURB-65 score >3 compared to the partially vaccinated group (63% vs. 53%, *P*=0.55). In addition, partially vaccinated patients exhibited increased rates of ARDS (6% vs 1%, P=0.61), septic shock (11% vs 3%, *P*=0.37), intubation (16% vs 8%, *P*=0.20) and mortality (21% vs 7%, *P*=0.09). Fully vaccinated patients exhibited lower rates of ICU admission compared to partially vaccinated patients (40% vs 15%, *P*=0.04). After adjusting for confounders, fully vaccinated patients had significantly lower odds of ICU admission (OR: 0.28, 95%CI: 0.15, 0.56) and intubation (OR: 0.22, 95%CI: 0.06, 0.81). However, fully vaccinated individuals had more in-hospital cardiac events (12% vs. 1%, *P*=0.61) and a lower odds of in-hospital mortality (OR: 0.18, 95%CI: 0.02, 1.66), but these were not statistically significant.

Multivariable logistic regression analysis for in-hospital mortality among fully and partially vaccinated patients with COVID-19 CAP

**Figure:**
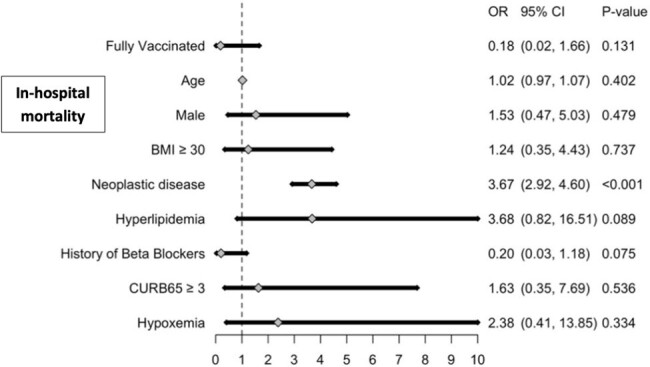

**Conclusion:**

This study highlights full vaccination's importance in reducing COVID-19 severity among hospitalized patients. Male gender and cancer were associated with higher mortality rates. Fully vaccinated patients had lower odds of ICU admission and intubation compared to partially vaccinated counterparts. Being fully vaccinated was not found to be associated with less cardiac events or mortality. These findings emphasize the need for comprehensive vaccination strategies and targeted public health initiatives.

**Disclosures:**

All Authors: No reported disclosures

